# Local versus distal acupoint low-level light therapy for pain relief in primary dysmenorrhea: protocol for a randomized, assessor- and statistician-blinded, pilot study

**DOI:** 10.3389/fmed.2026.1793534

**Published:** 2026-05-19

**Authors:** Jihye Kim, Minji Kim, Ae-Ran Kim, Bok-Nam Seo, Mi Hong Yim, Sunmi Choi

**Affiliations:** 1 Digital Health Research Division, Korea Institute of Oriental Medicine, Daejeon, Republic of Korea; 2 Clinical Research Coordinating Team, Korea Institute of Oriental Medicine, Daejeon, Republic of Korea; 3 School of Korean Convergence Medical Science, University of Science and Technology, Daejeon, Republic of Korea; 4 Korean Medicine Data Division, Korea Institute of Oriental Medicine, Daejeon, Republic of Korea

**Keywords:** acupoint phototherapy, Korean medicine, low-level light therapy, multimodal biosignal, primary dysmenorrhea, randomized controlled trial

## Abstract

**Background:**

Primary dysmenorrhea (PD) is a common gynecological condition affecting a substantial proportion of women of reproductive age. Non-steroidal anti-inflammatory drugs often provide incomplete pain relief and may be associated with adverse effects (AEs), highlighting the need for low-risk, non-pharmacological alternatives. Low-level light therapy (LLLT) has emerged as a promising non-invasive modality for pain management; however, the comparative effects of LLLT applied to local versus distal acupoints remain unclear.

**Method:**

This is a single-center, prospective, randomized, assessor- and statistician-blinded, parallel-group pilot study. A total of 60 women with moderate-to-severe PD will be randomized to receive LLLT at either local or distal acupoints. Interventions will be administered once daily for 7 consecutive days prior to menstruation across three menstrual cycles. Pain intensity will be assessed using the visual analog scale (VAS). Multimodal biosignals, such as electrocardiography, electromyography, electrodermal activity, photoplethysmography, respiration, and skin temperature, will be recorded to explore physiological responses to treatment. The primary outcome is the change from baseline in VAS measured at the scheduled post-menstrual visit occurring within 3 days after the onset of menstruation, assessed across three menstrual cycles. The secondary outcomes include biosignal changes, patient-reported outcomes, and the Patient Global Impression of Change.

**Conclusion:**

To the best of our knowledge, this will be one of the first randomized studies to directly compare LLLT applied to local and distal acupoints in patients with PD. As a comparative pilot study, it is not designed to establish definitive efficacy but to explore differences between stimulation strategies and to generate preliminary data for future confirmatory studies.

**Study protocol registration:**

https://cris.nih.go.kr/cris/search/detailSearch.do?seq=30497&sea. This study has been registered with the Clinical Research Information Service, identifier KCT0010827 (registration date: 29 July 2025).

## 1 Introduction

Primary dysmenorrhea (PD) is among the most prevalent gynecological complaints, affecting approximately 45–95% of women of reproductive age worldwide (1, 2). It is characterized by recurrent spasmodic lower abdominal pain during menstruation in the absence of identifiable pelvic pathology (3). Given its high prevalence and recurrent nature, PD imposes a substantial public health burden, significantly affecting daily activities, academic or work performance, and quality of life. Up to one-third of affected individuals report absenteeism or severe functional limitations during menstruation (1, 4). Accordingly, PD is increasingly recognized as a chronic pain condition with meaningful psychosocial and economic implications.

Current first-line treatments primarily include non-steroidal anti-inflammatory drugs (NSAIDs), which target prostaglandin-mediated uterine hypercontractility and ischemia (3, 5). However, approximately 20–25% of patients experience inadequate pain relief, and long-term use may be limited by gastrointestinal, renal, and cardiovascular adverse effects. Hormonal contraceptives represent an alternative option but may not be acceptable for individuals seeking fertility preservation or preferring non-hormonal approaches (6). These limitations have led to growing interest in safe, non-pharmacological interventions with improved tolerability and patient acceptability.

Low-level light therapy (LLLT), also referred to as photobiomodulation, has emerged as a promising non-invasive modality for pain management (7). Experimental and clinical studies have suggested that LLLT modulates cellular activity, improves microcirculation, and exerts anti-inflammatory and analgesic effects (7, 8). In PD, these mechanisms may help alleviate uterine ischemia and reduce smooth muscle hypercontractility. Preliminary clinical studies have reported reductions in menstrual pain following light therapy applied to the abdominal region, supporting its potential therapeutic role (9).

In traditional Korean medicine (TKM), acupoint-based interventions have long been used to regulate gynecological function and relieve menstrual pain. Local acupoints, such as CV4 (Guanyuan) and CV6 (Qihai), are traditionally associated with uterine regulation, whereas distal acupoints, including SP6 (Sanyinjiao), ST36 (Zusanli), and LI4 (Hegu), are believed to exert systemic regulatory effects (10, 11). With advances in medical technology, light-based stimulations have been increasingly applied to acupoints, integrating traditional meridian theory with modern photobiomodulation approaches. Compared with needle acupuncture, acupoint-based light therapy offers advantages such as non-invasiveness, standardization, and improved patient acceptability, supporting its potential as an electroceutical intervention in integrative gynecology (12).

Despite growing clinical interest, previous randomized controlled trials (RCTs) have primarily evaluated LLLT in comparison with sham or standard care, focusing on efficacy outcomes (13–15). However, key treatment parameters, particularly the optimal selection of stimulation sites, remain insufficiently understood. It remains unclear whether targeting local uterine-associated acupoints or distal systemic acupoints results in differential therapeutic outcomes in PD. This gap is clinically relevant, as both approaches are commonly used in practice but may involve distinct mechanisms, including local physiological modulation and systemic or central pain regulation.

Therefore, this study was designed as a comparative pilot randomized trial to explore potential differences between local and distal acupoint stimulation, rather than to establish efficacy. The primary objective is to generate preliminary data to inform treatment optimization and the design of future confirmatory studies. Multidimensional outcomes, including psychosocial and quality-of-life measures, will also be assessed to better capture the broader impact of PD.

## 2 Methods and analysis

### 2.1 Aim

This study aimed to compare the effects of LLLT applied to local versus distal acupoints on menstrual pain in women with PD.

### 2.2 Study design

This study is a randomized, assessor- and statistician-blinded, two-arm, parallel-group pilot study designed in accordance with the Standard Protocol Items: Recommendations for Interventional Trials (SPIRIT) 2025 statement (16). The results of the study will be reported in accordance with the Consolidated Standards of Reporting Trials (CONSORT) 2010 guidelines (17).

**Figure 1:**
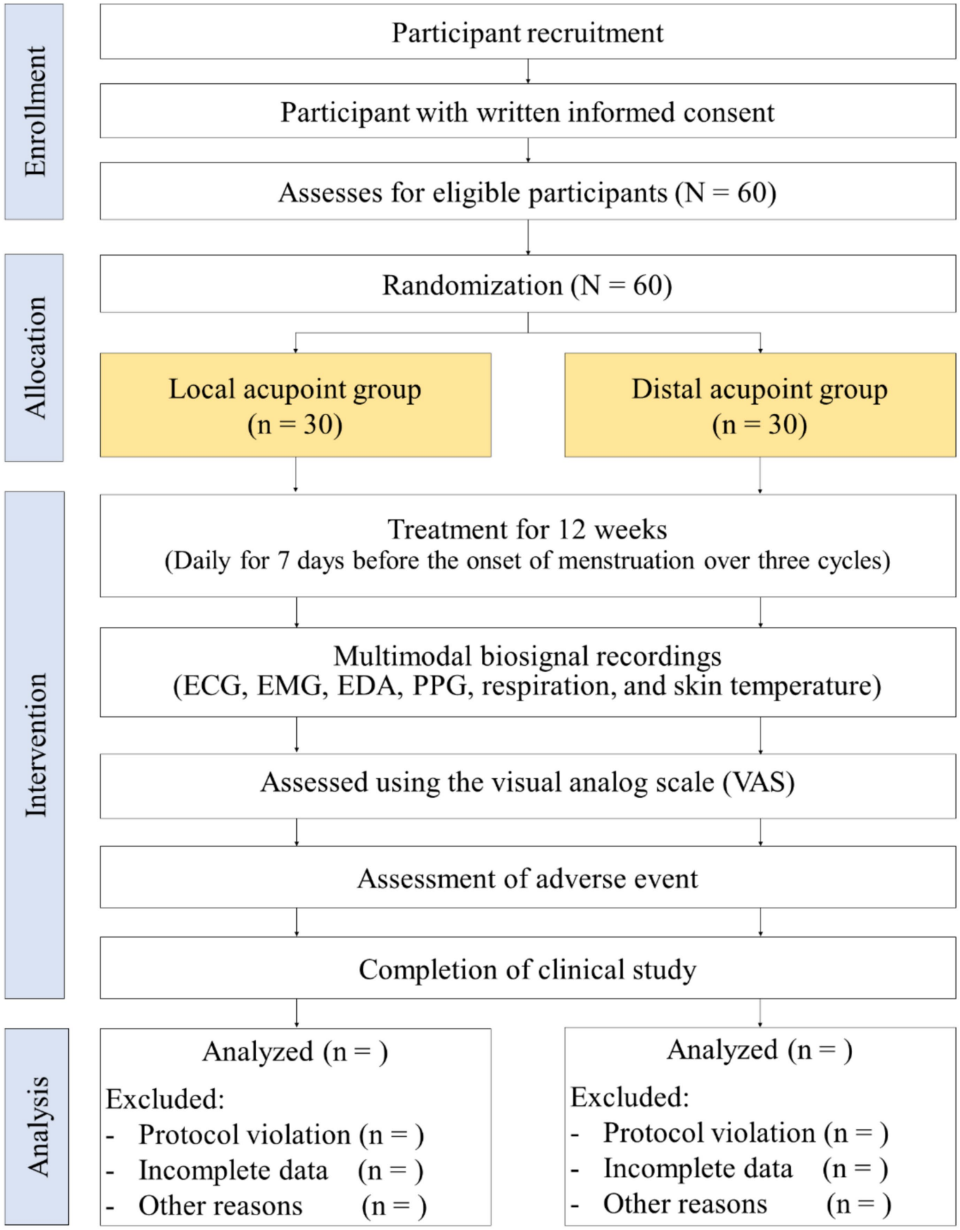
Study design flowchart. Flow diagram of the RCT comparing the local acupoint group and the distal acupoint group in 60 patients with PD. Participants were randomly assigned to receive low-level light therapy at either local or distal acupoints.

Participants who provide written informed consent will undergo eligibility screening, and a total of 60 participants will be enrolled for a 12-week study period (Figure 1).

This study adopts a comparative design without a sham or usual-care control group, as its primary objective is to explore differences between intervention strategies rather than to evaluate absolute efficacy.

The study protocol will adhere to the Declaration of Helsinki and Good Clinical Practice guidelines and has been approved by the Institutional Review Board of Woosuk University Jeonju Korean Medicine Hospital [Version 1.0, Reference Number: Institutional Review Board of Woosuk University Jeonju Korean Medicine Hospital (WSOH IRB) M2507-01]. All study procedures will be conducted at the Woosuk University Jeonju Korean Medicine Hospital in South Korea.

Any important modifications to the study protocol will be submitted to the IRB for approval prior to implementation and will be communicated to relevant parties, including investigators and trial registries, as required.

Written informed consent will be obtained from all participants by trained research personnel prior to enrollment. Participants will be provided with detailed information about the study procedures, potential risks, and benefits.

Recruitment is scheduled to begin in October 2025 and is expected to be completed by October 2026. Data collection and analysis are anticipated to be completed in the first half of 2027. Participants will be allocated in a 1:1 ratio to either the local or distal acupoint group. To assess effectiveness and safety, participants will attend visits at the onset of menstruation across three menstrual cycles. Detailed study procedures are presented in Table 1.

**Table 1:** Schedule of enrollment, interventions, and assessments in accordance with the SPIRIT statement.

Timepoint	Enrollment	Allocation	Post-allocation					Close-out
	Screening	Run-in period	Cycle 1 (4 weeks)		Cycle 2 (8 weeks)		Cycle 3 (12 weeks)	
Visit (Visit window)	Visit 0 (−2 weeks ± 2 days)	Visit 1 (−1 week ± 2 days)	1st self-treatment (7 days)	Visit 2 (menses + 3 days)	2nd self-treatment (7 days)	Visit 3 (menses + 3 days)	3rd self-treatment (7 days)	Visit 4 (menses + 3 days)
Enrollment								
Informed consent	●							
Demographics	●							
Menstrual history	●							
Medical and treatment history (including medication use)	●							
Physical examination	●							
Vital signs	●	●		●		●		●
Anthropometrics	●							
Laboratory tests (blood)^a^	●							
Pregnancy test	●							
Visual analog scale (VAS) for pain	●	●		●		●		●
Beck Depression Inventory-II (K-BDI-II)	●	●		●		●		●
Inclusion/exclusion criteria	●							
Allocation								
Randomization		●						
Intervention^b^								
Home-based LLLT at local acupoint (self-administration, daily for 7 days premenstrual)			●		●		●	
On-site LLLT at local acupoint				●		●		●
Home-based LLLT at distal acupoint (self-administration, daily for 7 days premenstrual)			●		●		●	
On-site LLLT at distal acupoint				●		●		●
Assessment								
Quality of life (EQ-5D-5L)		●		●		●		●
Stress response inventory-short form (SRI-SF)		●		●		●		●
State–trait anxiety inventory (STAI)		●		●		●		●
Device dispensing and training for home-based self-administration		●						
Clinical device return								●
Analgesic use check (type and dose)		●		●		●		●
Menstrual diary (distribution/collection)		●		●		●		●
Multimodal biosignal measurements^c^				●		●		●
Compliance with home-based LLLT				●		●		●
Patient Global Impression of Change (PGIC)								●
Changes in medical/treatment history		●		●		●		●
Adverse events				●		●		●
End of study/case closure								●

^a^If laboratory test results within 2 weeks prior to screening are available, they may be used in place of repeat testing at screening. Tests include complete blood count (CBC), liver function tests [such as total bilirubin, total protein, albumin, aspartate transaminase (AST), alanine transaminase (ALT), alkaline phosphatase (ALP), and gamma-glutamyl transferase (GGT)], renal function (such as BUN and creatinine), thyroid function (TSH), and HbA1c. ^b^Low-level light therapy will be performed as home-based self-administration once daily for 20 min during the 7 days prior to menstruation, followed by a single on-site supervised session at each cycle visit. Participants will record light therapy adherence and pain levels in the provided diary. ^c^Multimodal biosignal measurements (on-site only: pre−/during/post-stimulation). Biosignal monitoring will include six parameters: respiration (RSP), electrocardiogram (ECG), photoplethysmography (PPG), electrodermal activity (EDA), skin temperature (SKT), and electromyography (EMG). SPIRIT, Standard Protocol Items: Recommendations for Interventional Trials.

### 2.3 Participants

#### 2.3.1 Target disease

PD (ICD-10 N94.4) will be diagnosed based on clinical assessment, defined as recurrent menstrual pain in the absence of known pelvic pathology. Eligible participants must report a consistent history of dysmenorrhea over the previous 3 months and have a regular menstrual cycle. Routine imaging studies, such as ultrasound, will not be performed unless clinically indicated to exclude secondary causes.

To reduce clinical and physiological heterogeneity, relatively restrictive eligibility criteria were applied. Given the exploratory nature and small sample size of this pilot study, minimizing variability was considered important to improve the interpretability of between-group comparisons. While this approach may limit generalizability, it was deemed appropriate to enhance internal validity and is consistent with methodological recommendations for pilot studies emphasizing feasibility and preliminary effect estimation rather than definitive hypothesis testing (18).

Furthermore, participants will be clinically screened by investigators to exclude features suggestive of secondary dysmenorrhea, and individuals with suspected underlying pelvic pathology will not be enrolled.

The detailed inclusion and exclusion criteria are as follows:

#### 2.3.2 Inclusion criteria

Women aged 19–45 years with regular menstrual cycles and diagnosed with moderate-to-severe PD [visual analog scale (VAS) ≥ 40 mm for at least 3 cycles] will be included (19, 20). The exclusion criteria are secondary dysmenorrhea, chronic pelvic pain, use of hormonal contraceptives, or ongoing light-based therapy.

The inclusion criteria are as follows: (1) women aged 19–45 years; (2) individuals who have consistently experienced PD (menstrual pain) over the past 3 months, with a menstrual pain VAS score of 40 mm or higher; (3) individuals with a regular menstrual cycle ranging from 21 to 35 days (28 ± 7 days) over the past 3 months; and (4) individuals who have voluntarily provided written informed consent to participate in the clinical study.

#### 2.3.3 Exclusion criteria

The exclusion criteria were grouped into the following categories:

Psychiatric conditions or related medication use: participants were excluded if they had a history of major psychiatric disorders within the past 2 years, were currently using psychotropic medications including antidepressants, or were suspected of having moderate-to-severe depression based on the Korean version of the Beck Depression Inventory-II score of ≥23.

Gynecologic, reproductive, or hormone-related factors: participants were excluded for use of oral contraceptives or other hormone therapy within 3 months before screening; structural abnormalities that may cause dysmenorrhea, including uterine fibroids, ovarian cysts, endometriosis, or cervical stenosis; treatment for uterine inflammatory conditions, including endometritis or cervicitis, within 2 months before screening; use of an intrauterine contraceptive device; or pregnancy, breastfeeding, planned pregnancy, or potential pregnancy without medically acceptable contraception during the study period.

Clinically significant medical conditions or abnormal screening results: participants were excluded for thyroid disease or abnormal thyroid function, defined as thyroid-stimulating hormone level of ≤0.26 or ≥4.3 μIU/mL; severe hepatic or renal impairment or abnormal laboratory findings, including aspartate aminotransferase or alanine aminotransferase of ≥2 × the upper limit of normal [ULN], blood urea nitrogen of ≥2 × ULN, or creatinine of ≥2.0 mg/dL; malignancy within the past 5 years; hemoglobin of <11 g/dL or current treatment for anemia; uncontrolled hypertension, defined as systolic blood pressure of ≥180 mmHg or diastolic blood pressure of ≥110 mmHg; uncontrolled diabetes mellitus, defined as hemoglobin A1c of ≥9%; or any uncontrolled serious systemic disorder or infectious disease, including cardiovascular, respiratory, gastrointestinal, genitourinary, neurological/psychiatric, hematological, endocrine, or autoimmune diseases.

Device-related or procedural limitations: participants were excluded if they had known hypersensitivity or allergic reaction to the investigational medical device or if they were unable to self-administer the device as required because of compliance issues or other limitations.

Other reasons: participants were excluded if they had participated in another clinical study involving investigational medical devices or drugs for pain relief within 3 months before screening or if they were deemed unsuitable for the study by the investigator for any other reason.

#### 2.3.4 Analgesic handling

The use of rescue analgesics (such as NSAIDs) will be permitted as needed and will be systematically recorded, including timing, frequency, and dosage. Analgesic use within a predefined time window prior to VAS assessment will be included as a covariate in the primary analysis to account for its potential immediate effect on pain reporting.

All medication use will be recorded throughout the study period. In addition, cumulative analgesic use during the assessment period will be considered in sensitivity analyses.

Participants will be instructed to refrain from receiving other treatments for dysmenorrhea, including additional light therapy, transcutaneous electrical nerve stimulation, acupuncture, or other physical or complementary therapies, during the trial period.

#### 2.3.5 Methods of recruitment

To recruit participants for the clinical study, advertisements will be placed in the mass media, including flyers and daily newspapers, as well as on bulletin boards and websites of the participating institutions. If recruitment is delayed, additional local advertisements will be implemented through subways, busses, apartment bulletin boards, clinical study websites, and applications. All recruitment methods were approved by the IRB and pre-specified in the study registration.

### 2.4 Intervention and comparisons

The intervention will be conducted using a two-phase approach. During the premenstrual period (7 days prior to the onset of menstruation), participants will perform daily LLLT through home-based self-administration after receiving standardized training and device instructions. The same intervention protocol, including device settings and application procedures, will be applied during both home-based and on-site administration to ensure consistency of treatment delivery.

The home-based phase is intended to provide repeated therapeutic exposure, whereas each on-site visit is designed as a standardized assessment session under controlled conditions, including biosignal acquisition.

At each menstrual onset, participants will attend a scheduled on-site visit, where LLLT will be administered under investigator supervision. During these visits, biosignals will be collected before, during, and after stimulation to assess physiological responses to the intervention.

In both groups, LLLT is delivered simultaneously to the designated acupoints during each 20-min session. To ensure comparability between the groups, the intervention protocols were designed to provide equivalent dosing. In both groups, stimulation is applied to two acupoints per session, with identical treatment duration (20 min), identical device settings (including wavelength and power density), and comparable irradiation area per acupoint. Consequently, the energy delivered per acupoint and the total energy delivered per session are equivalent across the groups.

Biosignal assessments are conducted as exploratory outcomes under standardized on-site conditions.

#### 2.4.1 Local acupoints group (abdominal, near the uterus area)

**Figure 2:**
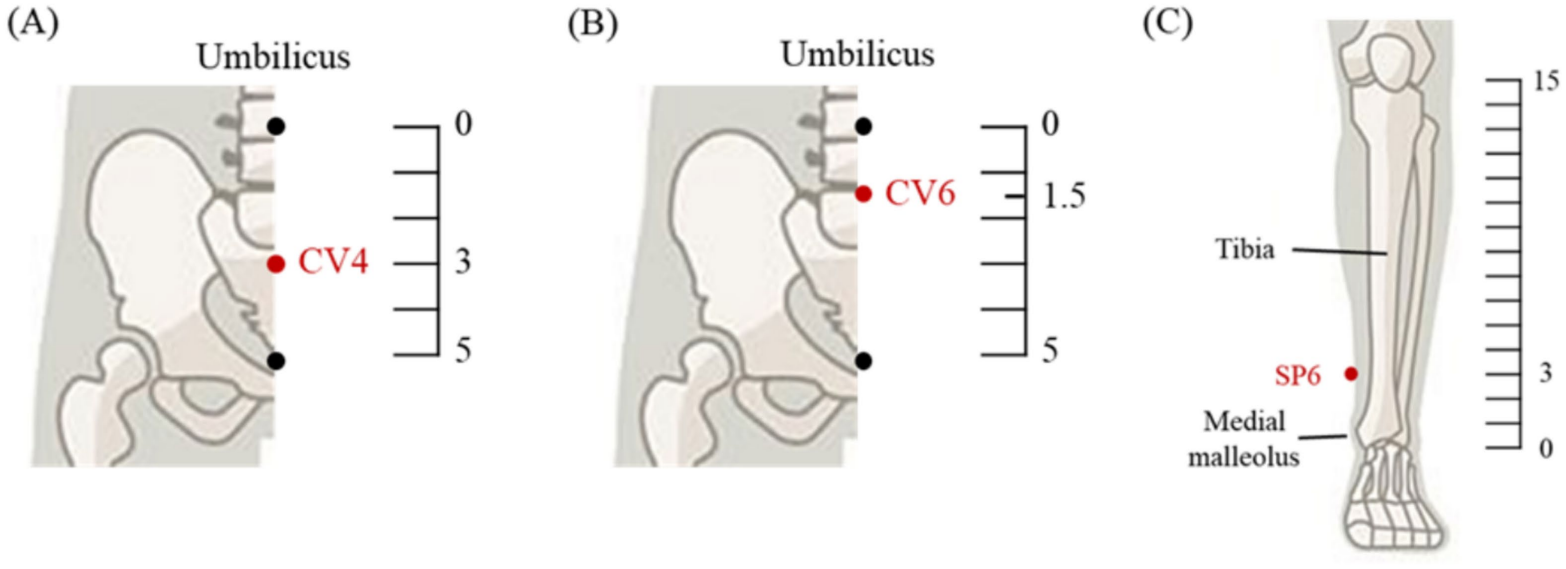
Selected acupoints of local group **(A,B)** and distal group **(C)**.

LLLT will be applied to the local acupoints CV4 (Guanyuan, “Gate of Origin”) and CV6 (Qihai, “Sea of Qi”) in the Conception Vessel. CV4 is located on the anterior midline of the lower abdomen, 3 cun inferior to the umbilicus, while CV6 is located 1.5 cun inferior to the umbilicus on the same line (Figures 2A,B). These acupoints are traditionally used to regulate menstruation and alleviate gynecological and lower abdominal pain (15, 21, 22). They were selected based on their widespread clinical use in the treatment of dysmenorrhea. Each session will last for a total of 20 min, with LLLT applied at CV4 and CV6, using a device emitting 610-nm red light. The intervention will be administered daily for 7 days prior to menstruation across three cycles.

#### 2.4.2 Distal acupoint group (lower limb, systemic regulation)

LLLT will be applied to the distal acupoint SP6 (Sanyinjiao, “Three Yin Intersection”). SP6 is located on the medial aspect of the lower leg, 3 cun above the medial malleolus and posterior to the medial border of the tibia (Figure 2C). This acupoint is traditionally associated with the regulation of menstruation and relief of lower abdominal pain and is widely used in gynecological practice, particularly for dysmenorrhea (23, 24). It was selected as the representative distal acupoint in this study. In the distal group, each session will last for 20 min, during which bilateral LLLT will be delivered to both left and right SP6 acupoints, resulting in stimulation at two acupoints per session, using a device emitting 610-nm red light. The intervention will be administered daily for 7 days prior to menstruation across three cycles.

#### 2.4.3 Interventional device

The interventional device to be used in this study is the Color DNA-WSF (Color Seven Co., Ltd., Seocho-gu, Seoul, South Korea), which has obtained authorization under the Korea Good Manufacturing Practice (KGMP, No. 3491) (25). This interventional device has been approved as a medical device by the Ministry of Food and Drug Safety (approval no.: 12–1,367, 26 October 2012).

The technical specifications of the device are as follows:

1. Operating time: 20 min per session
2. Output wavelength: 610 nm ± 10%
3. Output power density: 1.8 mW/cm^2^ ± 20%
4. Output mode: continuous wave
5. Irradiation area (hole size): Ø 4.4 mm ± 10%

This device will be used to deliver LLLT at specified acupoints according to the study protocol.

In both groups, each treatment session lasts for 20 min and is administered once daily for 7 consecutive days prior to the expected onset of menstruation across three menstrual cycles. In the local acupoint group, LLLT is applied to CV4 and CV6, whereas in the distal acupoint group, LLLT is applied bilaterally to SP6 using a 610-nm red light-emitting device.

In both groups, LLLT is delivered simultaneously to two designated acupoints during each 20-min session. Each acupoint is irradiated using an independent light source with identical output parameters, such as wavelength, power density, and irradiation area. Given that energy delivery is determined by power density, irradiation area, and exposure time, and these parameters are identical for each acupoint, the energy delivered per acupoint is equivalent across the groups.

As both groups receive simultaneous stimulation at two acupoints with identical device settings and treatment duration, the number of irradiation sites, per-acupoint dose, and total energy delivered per session are matched. This design minimizes potential confounding due to differences in dose distribution and enables a more direct comparison of stimulation location (local versus distal).

Participants are informed that the perceived sensation of stimulation may vary depending on individual sensitivity. To promote adherence, all treatment sessions are scheduled in advance, and participants receive reminders prior to each visit. Adherence is monitored by recording attendance at each session and documenting completion of the assigned treatment according to the study protocol.

### 2.5 Multimodal biosignal acquisition

Biosignals will be measured during three consecutive periods at each hospital visit: a 5-min baseline recording prior to light stimulation, a 20-min recording during the light therapy session, and a 5-min recording immediately after the intervention.

Biosignals will be collected using a system consisting of a biosignal acquisition unit, a laptop computer, and dedicated software. The biosignal acquisition unit is designed to simultaneously record six physiological signals: respiration (RSP), electrocardiogram (ECG), photoplethysmography (PPG), electrodermal activity (EDA), skin temperature (SKT), and electromyography (EMG). Each signal will be measured through specific acquisition modules connected to the MP160 data acquisition system (BIOPAC Systems Inc., Goleta, CA, United States). Wireless transmission will be supported by three Bionomadix® wireless units (BIOPAC Systems Inc., Goleta, CA, United States), which allowed for real-time data transfer to the laptop computer. Data collection and management will be performed using AcqKnowledge® software (BIOPAC Systems Inc., Goleta, CA, United States).

### 2.6 Randomization, allocation, and blinding

Participants who meet the eligibility criteria will be randomly assigned in a 1:1 ratio to either the local group (LLLT at CV4 and CV6) or the distal group (LLLT at bilateral SP6).

The random allocation sequence will be generated by an independent statistician using a computer-based random number generator in R software (R Foundation for Statistical Computing, Vienna, Austria) without stratification. A restricted randomization method with block randomization will be used. To minimize predictability, the block size will not be disclosed and will be kept in a separate document inaccessible to investigators responsible for participant enrollment and assignment.

Allocation concealment will be ensured using sequentially numbered, opaque, and sealed envelopes prepared by a researcher not involved in participant recruitment or intervention assignment. Investigators responsible for enrolling participants and assigning interventions will not have access to the random allocation sequence.

Due to the distinct anatomical locations of the interventions, participant blinding is not feasible. Therefore, the study will be conducted with blinding of outcome assessors and statisticians, while clinicians delivering the intervention will not be blinded. Outcome assessments will be performed by independent assessors unaware of group allocation, and statistical analyses will be conducted using blinded group codes.

Emergency unblinding will be permitted only if a participant experiences severe, unrelieved pain or requests withdrawal. All instances of unblinding will be documented, and the participant will be withdrawn from the intervention.

Written informed consent will be obtained from all participants prior to enrollment, and the clinicians will administer the assigned intervention according to the group allocation.

### 2.7 Sample size

As this study is an exploratory pilot trial, the sample size was determined primarily based on feasibility considerations and the need to obtain preliminary estimates for a future confirmatory randomized controlled trial, rather than to test a definitive efficacy hypothesis. We therefore did not base the sample size on a formal power calculation. A total of 60 participants, with 30 participants per group, was considered appropriate for evaluating key feasibility parameters, including recruitment, adherence, retention, safety, and completion of outcome assessments. This sample size was also expected to provide preliminary estimates of treatment effect and variability, which will be used to inform the design and sample size calculation of a future adequately powered confirmatory trial. Allowing for an anticipated dropout rate of approximately 20%, the target enrollment was set at 60 participants in total.

### 2.8 Outcomes

#### 2.8.1 Primary outcome

The primary outcome is the change from baseline in menstrual pain intensity measured using a 0–100-mm VAS (26). The primary endpoint is defined as the change from baseline in VAS measured at the scheduled post-menstrual visit occurring within 3 days after the onset of menstruation, assessed across three menstrual cycles.

A visit window of up to 3 days after menstruation onset is allowed to accommodate inter-individual variability in the timing of peak pain and to ensure feasibility while minimizing protocol deviations. Assessments will be conducted at baseline and at each post-menstrual visit (visit window: day 1 + 3 days) over three menstrual cycles.

To further characterize individual pain patterns, VAS scores will be recorded daily from 7 days prior to menstruation through the first 3 days of menstruation. Sensitivity analyses will be conducted using alternative outcome definitions, including the maximum VAS during menstrual days 1–3.

To evaluate treatment effects, a linear mixed-effects model (LMM) will be used, with treatment group, visit (time point), and group-by-visit interaction specified as fixed effects, and participant-level variability included as a random effect. Baseline VAS will be included as a covariate (27).

#### 2.8.2 Secondary outcomes

The secondary outcomes include changes in biosignals and patient-reported outcomes, such as the Korean version of the Beck Depression Inventory-II (K-BDI-II) (28), the EuroQol 5-Dimension 5-Level (EQ-5D-5L) (29), the Stress Response Inventory-Short Form (SRI-SF) (30, 31), and the State–Trait Anxiety Inventory (STAI) (32) assessed at baseline and at each post-menstrual visit (visit window: day 1 + 3 days) over three cycles. PGIC will be assessed once at the final visit (33).

Multimodal biosignals: Multimodal biosignals will be acquired at baseline and at each post-menstrual visit over three menstrual cycles, including RSP, ECG, PPG, EDA, SKT, and EMG. Biosignal measurements will be analyzed as exploratory outcomes. The underlying hypothesis is that stimulation at different acupoint locations may induce differential modulation of physiological responses. Given the exploratory nature of these analyses, no formal adjustment for multiple comparisons will be applied, and the findings will be interpreted as hypothesis-generating.

K-BDI-II: The K-BDI-II is the validated Korean translation of the Beck Depression Inventory-II, a widely used self-reported measure of depressive symptoms. It consists of 21 items, each scored on a 4-point Likert scale (0–3), reflecting symptom severity over the past 2 weeks. Total scores range from 0 to 63, with higher scores indicating more severe depressive symptoms. Standard cutoff scores are defined as minimal (0–13), mild (14–19), moderate (20–28), and severe (29–63).

EQ-5D-5L: The EQ-5D-5L is a standardized instrument for assessing health-related quality of life. It comprises five dimensions: mobility, self-care, usual activities, pain/discomfort, and anxiety/depression. Each dimension is rated on five levels (from no problems to extreme problems). Responses are converted into a single index score using country-specific value sets, with higher scores indicating better health status. In addition, the EQ-5D-5L includes a visual analog scale (VAS; 0–100) for self-rated overall health.

SRI-SF: The SRI-SF is a validated short-form instrument used to assess multidimensional stress responses. It consists of 22 items scored on a 5-point Likert scale (0–4), covering emotional, somatic, cognitive, and behavioral domains. Total scores range from 0 to 88, with higher scores indicating greater stress response levels.

STAI: The STAI is a self-report measure used to evaluate anxiety. It consists of 40 items divided into two subscales: state anxiety (20 items), assessing transient anxiety, and trait anxiety (20 items), measuring general anxiety disposition. Each item is rated on a 4-point Likert scale, with subscale scores ranging from 20 to 80. Higher scores indicate greater levels of anxiety.

PGIC: The PGIC is a single-item measure assessing a patient’s overall perception of change following treatment. It is rated on a 7-point Likert scale ranging from 1 (significantly improved) to 7 (worse response). It is widely used as a global measure of perceived treatment effectiveness.

### 2.9 Statistical analysis

#### 2.9.1 Analysis populations

The analysis populations will consist of the full analysis set (FAS) and the per protocol set (PPS). The FAS will include all randomized participants who receive at least one application of the investigational device and have at least one post-baseline assessment and will be used for primary efficacy analyses in accordance with the intention-to-treat (ITT) principle. The PPS will include participants without major protocol deviations and with a treatment compliance rate of at least 70% and will be used for supportive analyses.

Missing data will be handled within the LMM and generalized linear mixed-effects model (GLMM) using maximum likelihood estimation under the missing-at-random assumption, which allows valid inference without explicit imputation.

In addition, sensitivity analyses will be conducted using multiple imputation to assess the robustness of the results to different missing data assumptions.

No interim analyses are planned.

#### 2.9.2 Demographic data and baseline clinical characteristics

Demographic variables will include age, height, weight, smoking status, alcohol consumption, physical activity, and educational level. Baseline clinical characteristics will include relevant medical history, concomitant medications, baseline VAS scores, and vital signs.

Continuous variables will be summarized as mean ± standard deviation, and categorical variables will be summarized as frequencies and percentages. Between-group comparisons will be performed using the independent *t*-test or Wilcoxon rank-sum test, depending on the distribution of the data. Categorical variables will be compared using the chi-square test or Fisher’s exact test, as appropriate.

#### 2.9.3 Between-group comparisons

The primary endpoint, defined as the change from baseline in the VAS measured at the scheduled post-menstrual visit occurring within days 1–3 after menstruation onset, will be analyzed using an LMM. Fixed effects will include treatment group, visit (time point), and the group-by-visit interaction, with baseline VAS included as a covariate. Analgesic use will also be included as a covariate to adjust for its potential confounding effect on pain outcomes. Analgesic exposure in the primary analysis will be defined based on use within a predefined time window prior to VAS assessment. Cumulative analgesic use during the assessment period will be considered in sensitivity analyses. Participant-level variability will be modeled as a random effect.

For secondary outcomes, continuous variables, such as biosignal measures and patient-reported outcomes, will be analyzed using LMMs, and binary or ordinal outcomes, such as PGIC, will be analyzed using the generalized linear mixed-effects models (GLMMs), with treatment group, visit, and group-by-visit interaction specified as fixed effects and participant-level variability as a random effect.

Biosignal analyses will be conducted as exploratory outcomes, and the findings will be interpreted as hypothesis-generating.

### 2.10 Data management and confidentiality

Data management for this study will be conducted by the Korea Institute of Oriental Medicine. All study-related documents will be assigned unique identification codes to ensure participant anonymity, and personally identifiable information will be excluded. Access to identification records will be restricted and permitted only with prior approval from the IRB. Trial conduct will be monitored by the research team to ensure adherence to the study protocol and data quality standards.

Clinical research coordinators will enter data into electronic case report forms (eCRFs), and an independent data manager will monitor data recording and management. All entered data will be verified by an investigator not involved in the clinical procedures, and data entry will be double-checked for accuracy.

The data will be securely stored in the iCReaT data management system, with access restricted by user authentication. Only the data manager and an independent statistician, who are not involved in the conduct of the trial, will have full access to the complete dataset. Each participating institution will have access only to its own data.

The central data management center will operate independently of the study sponsor to ensure neutrality. Unauthorized access to the data will not be permitted without IRB approval. All source data will be securely archived for 3 years after study completion. Written informed consent will be obtained from all participants for the use and sharing of anonymized data.

No formal data monitoring committee will be established for this study, as the trial is considered low-risk and involves non-invasive interventions.

No interim analyses or formal stopping guidelines are planned.

### 2.11 Safety assessment

All adverse events occurring in the two groups will be systematically recorded throughout the study period. Each AE will be evaluated in terms of severity, seriousness, relationship to the medical device, requirement for additional treatment, and outcome.

Participants who experience adverse events related to the study will receive appropriate medical care. Compensation for study-related harm will be provided in accordance with the institutional policies and applicable regulations.

### 2.12 Ethical considerations

This study will be conducted in accordance with the Declaration of Helsinki and Korean Good Clinical Practice guidelines. The protocol was approved by the Institutional Review Board of Woosuk University Jeonju Korean Medicine Hospital (WSOH IRB M2507-01) and registered with the Clinical Research Information Service (KCT0010827, July 29, 2025). Written informed consent will be obtained from all participants prior to enrollment. To ensure confidentiality, numerical codes will be used in place of personal identifiers, and all participant information will be securely protected. The study protocol (version 1.0, July 2025) specifies that recruitment will take place from October 2025 to October 2026.

## 3 Discussion

### 3.1 Rationale

PD remains one of the most prevalent gynecological conditions among women of reproductive age, with a substantial impact on quality of life and daily functioning. Although NSAIDs are widely used as first-line treatment, their effectiveness is limited in a proportion of patients, and long-term use may be constrained by adverse effects. These limitations have led to increasing interest in low-risk, non-pharmacological treatment strategies.

LLLT, or photobiomodulation, has emerged as a potential therapeutic option due to its non-invasive nature and proposed analgesic and anti-inflammatory effects. Although previous studies have suggested potential benefits of LLLT for menstrual pain, key treatment parameters, particularly stimulation site selection, remain insufficiently defined.

Within the framework of TKM, local and distal acupoints are believed to exert distinct therapeutic effects. However, direct comparisons between these approaches using LLLT are lacking. Accordingly, this study was designed as a comparative pilot trial to explore whether stimulation at local (CV4, CV6) versus distal (SP6) acupoints may result in differential clinical and physiological responses.

The study design ensured strict dose equivalence between the groups using independent light sources with identical output parameters for each acupoint, allowing a more direct comparison of local versus distal stimulation effects.

### 3.2 Overview of the study

To the best of our knowledge, this is one of the first randomized studies to directly compare LLLT applied to local and distal acupoints in women with PD. This study investigates whether stimulation site influences treatment outcomes by comparing local and distal acupoint-based LLLT in women with PD. In addition to pain intensity, multimodal biosignal measurements are included to explore potential physiological correlates of treatment response. These analyses are exploratory and intended to generate hypotheses for future studies.

### 3.3 Strengths and limitations

This study has several strengths. It adopts a randomized design to compare two clinically relevant acupoint strategies and incorporates both subjective and objective outcome measures. Repeated interventions across multiple menstrual cycles allow evaluation of cumulative effects.

However, several limitations should be considered. This study is designed as a comparative study without a control group, which limits the ability to determine the absolute efficacy of LLLT and does not allow full control of the placebo effects. If both groups show minimal therapeutic benefits, the clinical interpretability of the comparison may be limited. Nevertheless, this design was intentionally selected to explore acupoint-specific effects and inform treatment optimization as a preliminary step toward future confirmatory studies. As a single-center pilot study with a relatively small sample size, the findings may have limited generalizability.

In addition, participant blinding was not feasible due to the distinct anatomical locations of the interventions. Given that the primary outcome is based on a subjective measure (such as VAS), this may introduce expectation and reporting bias. To mitigate this risk, outcome assessments were conducted by independent assessors, and statistical analyses were performed using blinded group allocation codes. The use of restrictive eligibility criteria may further limit the generalizability, and the focus on short-term outcomes precludes the assessment of long-term effects.

### 3.4 Implications and future directions

Despite these limitations, this pilot study is expected to provide valuable preliminary insights into the feasibility and potential clinical relevance of acupoint-targeted photobiomodulation in PD. The findings may inform the design of future randomized controlled trials incorporating appropriate control groups and contribute to the optimization of treatment strategies.
